# Cholera

**Published:** 1849-04

**Authors:** 


*Cholera.*—The packet ship *Liverpool*, from Liverpool, with four hundred passengers, recently arrived at New York, with cases of cholera on board, forty cases having proved fatal during the passage. The vessel and passengers were placed in quarantine, where they are still detained. No new cases, however, have lately been developed, nor has the disease made its appearance out of the vessel.

We have no information of the existence of cholera, at the present time, as an epidemic in any part of our country. Isolated cases are reported to occur occasionally at New Orleans, and other south-western towns, showing that there is not entire exemption from the specific cause, and, consequently, from the probability of the re-appearance of the disease in an epidemic form.

